# Lutein and Astaxanthin Supplementation Induce Competitive Inhibition of Carotenoid Deposition in Egg Yolk

**DOI:** 10.3390/ani15131869

**Published:** 2025-06-24

**Authors:** Xia Chen, Zhixun Yan, Bing Zhang, Lingchao Zeng, Urmita Chowdhury, Mohammad Hasanuzzaman Pabitra, Jing Cao, Zhipeng Wang, Yanghua He, Huagui Liu, Qin Chu

**Affiliations:** 1 Institute of Animal Husbandry and Veterinary Medicine, Beijing Academy of Agriculture and Forestry Sciences, Beijing 100097, China; chenxia_91@163.com (X.C.); yanzhixun2008@sina.com (Z.Y.); 13240150468@163.com (B.Z.); yfzjc@163.com (L.Z.); caojing2046555@163.com (J.C.); 2 College of Animal Science and Technology, Northeast Agricultural University, Harbin 150038, China; wangzhipeng@neau.edu.cn; 3 Department of Human Nutrition, Food and Animal Sciences, University of Hawai‘i at Mānoa, Honolulu, HI 96822, USA; urmita@hawaii.edu (U.C.); mpabitra@hawaii.edu (M.H.P.); yanghua.he@hawaii.edu (Y.H.)

**Keywords:** lutein, astaxanthin, egg quality, laying performance, competitive inhibition

## Abstract

Lutein- and astaxanthin-fortified eggs can greatly improve both nutritional and economic values of eggs. High doses of lutein and astaxanthin are often supplemented in hen diets to rapidly accumulate lutein and astaxanthin in eggs. In this study, an additional 100, 200, and 400 mg/kg lutein and astaxanthin were added in hen diets to explore the effects of high-dose lutein and astaxanthin on egg quality, egg production, and carotenoid deposition in eggs, which remains unclear. The results showed that high-dose lutein and astaxanthin supplementation had no significant impact on laying rates and egg quality, except for markedly improving yolk color. Additionally, a quantitative profile of carotenoids in egg yolk was performed through liquid chromatography–mass spectrometry. A total of 26 carotenoids were detected including 14 whose concentrations exceed 0.1 µg/g. Notably, the zeaxanthin content in yolk decreased with the increase in levels of lutein supplementation. Conversely, the content of lutein, zeaxanthin, 5,6 epoxy-lutein-caprate-palmitate in yolk decreased as astaxanthin supplementation increased. These findings suggest that high doses of lutein and astaxanthin supplementation may competitively inhibit the deposition of other carotenoids in egg yolk. Overall, this study suggests that during the production of functional eggs enriched with lutein and astaxanthin, it is important to consider the interaction effects among different carotenoids.

## Simple Summary

In Chinese poultry farming, high doses of lutein and astaxanthin (>100 mg/kg) may be added in feed to rapidly produce a “functional egg”. However, the effects of high-dose lutein and astaxanthin on egg quality, production, and carotenoid deposition are poorly understood. Additionally, carotenoid competitive absorption was observed in humans, yet the influence of dietary lutein and astaxanthin on the deposition of carotenoids in egg yolk is underexplored. This study investigates the effects of 100–400 mg/kg lutein and astaxanthin supplementation on egg quality, laying performance, and carotenoid deposition in yolks. Dietary high-dose lutein and astaxanthin had no negative effect on laying performance and egg quality but markedly improved yolk color. Additionally, dietary lutein and astaxanthin may induce carotenoid interactions. This study advances the understanding of carotenoid deposition and interaction in egg yolk, offering practical implications for producing carotenoid-fortified eggs.

## 1. Introduction

Carotenoids are known for their diverse biological functions, including antioxidant activity, eye health protection, immune system modulation, anti-inflammatory effects, cognitive function and neuroprotection [1,2]. Moreover, beyond nutritional benefits, carotenoids are also widely used in the poultry industry to enhance egg yolk color [3,4,5], a crucial factor influencing consumer preferences [6]. Yolk color, ranging from pale yellow to deep orange, is directly influenced by the type and content of carotenoids in a hen’s diet [7]. Consumer preferences for yolk color vary regionally. In Europe, yellow yolks are preferred, while pale or non-uniform colors are often associated with poor quality [8]. In contrast, Asian consumers increasingly associate special yolk colors with superior taste and high-value products [9]. Therefore, carotenoids are commonly added to poultry feed to produce ‘functional eggs’, which not only enhance nutritional value, but also greatly increase the economic value of eggs.

In nature, more than 1200 types of carotenoids have been identified [10]. These carotenoids are classified into two main categories based on their chemical structure and function: carotenes (e.g., α-carotene, β-carotene, lycopene) and xanthophylls (e.g., lutein, zeaxanthin, canthaxanthin, β-cryptoxanthin) [11]. Among these, lutein and astaxanthin are particularly important fat-soluble carotenoids with significant roles in human health. Lutein, a major component of the macular area of the retina, protects against light-induced macular impairment [12]. Astaxanthin, renowned as a natural super antioxidant, exhibits antioxidant activity several times stronger than that of carotene and vitamin C [13]. Meanwhile, as a yellow and red pigment respectively, lutein and astaxanthin are commonly used in the poultry industry to improve the color of egg yolks [14].

Over the past decades, numerous studies have investigated the effects of lutein and astaxanthin supplementation on egg quality and laying performance, with the lutein supplementation levels usually not exceeding 40 mg/kg and the astaxanthin supplementation levels being generally below 100 mg/kg [14,15,16,17,18,19]. In Chinese poultry farming practices, hen diets may be supplemented with high doses (>100 mg/kg) of lutein and astaxanthin. This practice can rapidly increase the content of lutein and astaxanthin in eggs. In this way, it not only quickly enhances the nutritional value of eggs but also brings considerable economic benefits to poultry farmers. However, the effects of high doses of lutein and astaxanthin supplementation on egg quality and egg production were poorly studied. Additionally, different carotenoids may have interactions during digestion, absorption, and tissue deposition. In humans, competitive interactions during absorption have been observed [20]. In chickens, competitive inhibition among lutein, zeaxanthin, and β-carotene has been reported in the plasma, retina, and other tissues [21]. Nevertheless, the influences of dietary lutein and astaxanthin supplementation on the deposition of other carotenoids in eggs has remained poorly understood. Therefore, it is necessary to explore the influences of high-dose lutein and astaxanthin addition on egg quality, egg production performance, and the deposition of carotenoids in egg yolk.

In this study, an additional 100, 200, and 400 mg/kg of lutein and astaxanthin were added in hen diets separately to explore the effects of high-dose lutein and astaxanthin on egg quality and egg production. Furthermore, we systematically examined the deposition of various carotenoids in egg yolk, providing new insights into the interaction effects between carotenoids in egg yolk.

## 2. Materials and Methods

### 2.1. Animals and Diets

A feeding study was conducted to investigate the influences of supplementing high-dose lutein and astaxanthin at three different levels on egg quality, egg production, and the deposition of carotenoids in eggs. Beijing-You chicken laying hens aged 28 weeks (*n* = 350) were randomly assigned to six treatment groups and a control group. In the control group, a corn–soybean basal diet was provided according to the nutrient recommendations of Beijing-You chickens (Table S1). For daily intake, the hens need to have their feed intake restricted to control body weight during the laying period, as required by the Beijing-You Chicken Feeding and Management Technical Code (DB11/T 1378—2023) [22]. Therefore, during the trial, each hen’s feed intake was restricted to 100 g per day. For the six treatment groups, an additional 100, 200, and 400 mg/kg lutein or astaxanthin was supplied, respectively, based on the same basal diet (Lutein_100, Lutein_200, Lutein_400, Asta_100, Asta _200, and Asta _400). The lutein powder (derived from marigold, containing 2% lutein) was purchased from the Chenguang Biotech Group Co., Ltd. (Shijiazhuang, Hebei, China), while the astaxanthin powder (derived from *Haematococcus pluvialis*, containing 2% astaxanthin) was provided by Yunnan Astalphy Biotech Co., Ltd. (Kunming, Yunnan, China). Hens were reared in a room where the environmental temperature was 21 ± 3 °C and the photoperiod was 16 h light followed by 8 h darkness. Hens were housed in 8 stacks × 3 tiers per stack, with one bird per cage.

### 2.2. Laying Performance and Egg Quality Measurement

After 1-week acclimation, a 7-week trial was carried out. During the trial, the number of chickens and eggs was recorded daily, from which the daily laying rate was calculated. On the 28th day of the trial, 30 fresh eggs per group were collected for egg quality evaluation. The egg sharp tester (FHK, Fujihira Industry Co., Ltd., Anjō, Japan) and eggshell force gauge (EFR-01, Robotmation, Tokyo, Japan) were used to measure egg shape index and eggshell strength, respectively. The eggshell color at the blunt, equator, and sharp was measured with a spectrophotometer (CM-700d, Konica Minolta, Japan). The eggshell thickness at the blunt, equator, and sharp was measured with an eggshell thickness analyzer (ETG-1016, Robotmation, Tokyo, Japan). The eggshell color and eggshell thickness for each egg were determined by averaging the values from the blunt, equator, and sharp. The egg quality was measured by an egg multi-tester (EMT-7300Ⅱ, Robotmation, Tokyo, Japan). Subsequently, yolks were separated with a yolk separator and five yolks were pooled together into one sample. Overall, six pooled samples were obtained per group, with three used for the measurement of lutein and astaxanthin and the other three for carotenoid profile analysis.

Additionally, another 30 fresh eggs per group were collected and weighed on the 29th day of the trial, which was represented as W1. Then, eggs were stored at 25 ± 1 °C for 30 days following a second weighing, represented as W2. Subsequently, the albumen height and Haugh units of the eggs were measured by egg multi tester (EMT-7300Ⅱ, Robotmation, Tokyo, Japan). The proportion of egg weight loss was calculated using the formula in Equation (1), as follows:

(1) $$Egg\;weight\;loss\;proportion\;(\%)=\frac{W1-W2}{W1}*100$$

### 2.3. Determination of Lutein and Astaxanthin Content in Egg Yolk

The content of lutein and astaxanthin in egg yolk was measured using the pooled yolks, with 3 pooled samples per group. Lutein was quantified according to the National Food Safety Standards (GB 5009.248-2016) [23]. Briefly, 2 g of yolk samples, 0.2 g of butylated hydroxytoluene (BHT), and 10 mL of ethanol were blended comprehensively. Subsequently, the mixture and 10% KOH (10 mL) were shaken for 30 min in a dark environment to facilitate saponification. After saponification, the mixture was extracted with 10 mL of extraction agent (composed of 1 g BHT, 200 mL cyclohexane, 400 mL diethyl ether, and 400 mL n-hexane). Subsequently, the mixture was centrifuged (4500 r/min) for 3 min. The extraction steps were repeated two times. The extraction solutions were combined, washed with 10 mL of water and centrifuged (4500 r/min, 3 min) to achieve phase separation. The washing was repeated one more time. The organic phases were combined and concentrated at room temperature until nearly dry. The residue was dissolved in 0.1% BHT ethanol solution (0.1 g BHT dissolved in 100 mL ethanol) to 25 mL and filtered by 0.45 µm membrane. Finally, 50 μL of the filtered extract was analyzed using liquid chromatography (Agilent, 1260 Infinity, Santa Clara, CA, USA). The analysis was performed using a C30 chromatographic column (5 µm, 30 °C, Agilent, CA, USA). The mobile phase is comprised of two solvents (methanol/water (88:12, *v*/*v*) with 0.01% BHT, and methyl tertiary butyl ether with 0.01% BHT) with the speed of 1.0 mL/min. The peak area at the wavelength of 445 nm was scanned. Subsequently, a standard curve was constructed by plotting the concentration of the working standard solution, which was employed to calculate the lutein concentration of the samples.

The content of astaxanthin was measured using the UV method. Approximately 2 g of egg yolk was fully dissolved with 5 mL of acetone. Then, it was centrifuged (4500 r/min) for 10 min and the supernatant was transferred to a brown volumetric flask. The extraction process was carried out until the supernatant turned colorless. The acetone was supplemented into the volumetric flask to 50 mL. The solution was filtered by a membrane (0.45 μm). The absorbance of the sample was measured at 478 nm using a UV-Vis spectrophotometer (Shimadzu, UV-2600, Kyoto, Japan) with acetone serving as the blank control. The content of astaxanthin was calculated according to the following formula:

(2) $$Astaxanthin\;content\;\left(\%\right)=\frac{A_{\left(478\right)}*V*a}{E*M}*100$$

where A _(478)_ is the absorbance of the sample at 478 nm, V is the volume of the solution, a is the dilution multiple, E is the mass extinction coefficient (2200) of a 1% astaxanthin acetone solution at 478 nm, and M is the sample weight (g).

### 2.4. Carotenoid Profile Analysis in Egg Yolk

To further confirm the relationships among different carotenoids, the composition and quantification of carotenoids in yolk were explored by LC-MS (QTRAP^®^ 6500+, SCIEX, Marlborough, MA, USA), with 3 samples per group. Initially, 50 mg of yolk sample and 0.5 mL mixture solution (n-hexane: acetone: ethanol = 1:1:1, *v*/*v*/*v*) with 0.01% BHT (g/mL) were mixed for 20 min and centrifuged (12,000 r/min). The supernatant was collected, and the residue was extracted again. The supernatant was dried with nitrogen. Then, the extract was reconstituted using 100 μL MeOH/MTBE (1:1, *v*/*v*) and filtered through a 0.22 μm filter membrane. For the LC-MS analysis, the following conditions were applied. A C30 chromatographic column with a particle size of 3 μm, an inner diameter of 2.0 mm and a length of 100 mm was utilized. The solvent system comprised two components: solvent A, which was a mixture of methanol and acetonitrile in a volume ratio of 1:3, supplemented with 0.01% BHT and 0.1% formic acid; and solvent B, methyl tert-butyl ether containing 0.01% BHT. Furthermore, scans were carried out using a triple quadrupole–linear ion trap mass spectrometer (QTRAP, SCIEX, Marlborough, MA, USA). This system was equipped with an APCI heated nebulizer operating in positive ion mode. The operational parameters for the APCI source were as follows: the ion source was designated as APCI+, the source temperature was set to 350 °C, and the curtain gas (CUR) was adjusted to 25.0 psi. Data acquisitions and carotenoid quantification were performed by Analyst software (version 1.6.3) and Multiquant software (version 3.0.3).

### 2.5. Statistical Analysis

For statistical analysis, R software (version 4.2.0) was used. One-way analysis of variance (ANOVA) was conducted with lutein and astaxanthin dose as the factor. The Tukey test was used for performing multiple comparisons among groups. Data visualization was also generated with the ggplot2 package in R (version 4.2.0). The descriptive statistics in tables are presented as mean ±  standard deviation with significant differences at probability level *p* < 0.05.

## 3. Results

### 3.1. Laying Performance and Egg Quality

**Figure 1:**
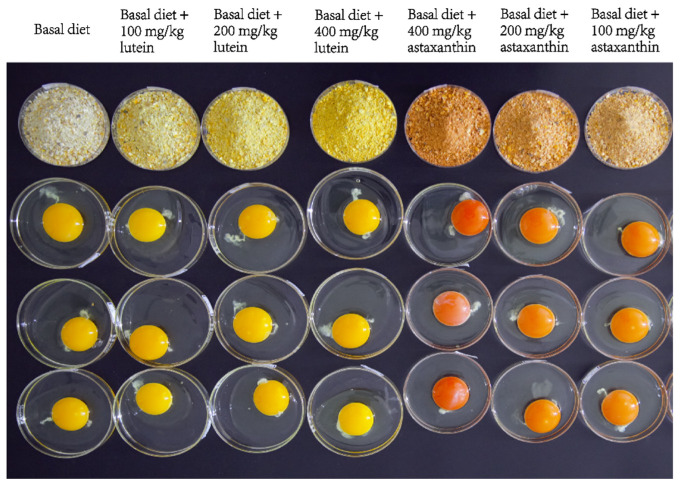
The appearances of feeds and eggs in the seven groups of this study.

Lutein and astaxanthin visually changed the coloration of both the feed and the egg yolk. Figure 1 shows the appearance of feed and eggs from the seven groups at the end of the fifth week of the trial. The yolk color of the control group was yellow, with a yolk score of 9.13 ± 0.60. The yolk of the lutein-supplemented groups exhibited a bright yellow hue, with yolk color scores ranging from 10.5 ± 1.02 to 11.4 ± 0.81 (*p* < 0.05, Table 1). For the astaxanthin-supplemented groups, the yolk color varied from orange to red, with yolk color scores ranging from 16.57 ± 0.74 to 18.01 ± 0.69. Notably, yolk color scores increased significantly as the levels of lutein and astaxanthin supplementation were raised. However, no significant differences were observed in eggshell strength, eggshell thickness, eggshell color (L*, a*, b*), egg shape index, egg weight, albumen height, and Haugh unit in the six lutein and astaxanthin treatment groups compared to the control group (*p* > 0.05).

**Figure 2:**
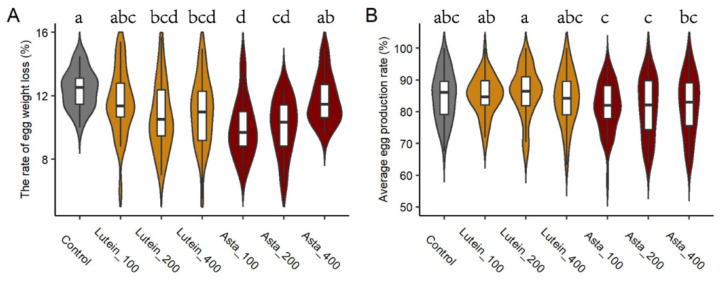
The effects of dietary lutein and astaxanthin supplementation on (**A**) egg storability and (**B**) laying performance. The box plot showed the key statistics: the median, quartiles, and outliers. The violin plot uses kernel density estimation (KDE) to show the probability density distribution of data. Control: basal diet; Lutein_100, Lutein_200, and Lutein_400: basal diet supplemented with 100, 200, and 400 mg/kg lutein, respectively. Asta_100, Asta_200, and Asta_400: basal diet supplemented with 100, 200, and 400 mg/kg astaxanthin, respectively. ^a–d^ Bars with different letters mean significantly different at *p* < 0.05.

The egg storability was also evaluated across the seven experimental groups (Table 2). After one month of storage, no significant differences were observed in albumen height and Haugh unit between the control group and treatment groups (Table 2, *p* > 0.05). However, the egg weight loss proportion in the Lutein_200 and Lutein_400 groups were significantly lower than those in the control group (Figure 2A, *p* < 0.05). Similarly, the Asta_100 and Asta_200 groups have lower egg weight losses than the control group (*p* < 0.05). While the egg weight loss proportion of the Lutein_100 group and Asta_400 group were not significantly different from that of the control group (*p* > 0.05).

Additionally, the effects of high-dose lutein and astaxanthin supplementation on laying performances were studied (Figure 2B). The laying performance of three lutein-supplemented groups were 85.21%, 86.07%, and 84.15% respectively, showing no significant difference with the control group (84.9%, *p* > 0.05). For astaxanthin-supplemented groups, although the laying rates (81.46%, 81.39%, and 82.09%) were lower than that of the control group, the differences did not reach the statistical significance (*p* > 0.05).

### 3.2. Astaxanthin Competitively Inhibits Lutein Deposition in Egg Yolk

**Figure 3:**
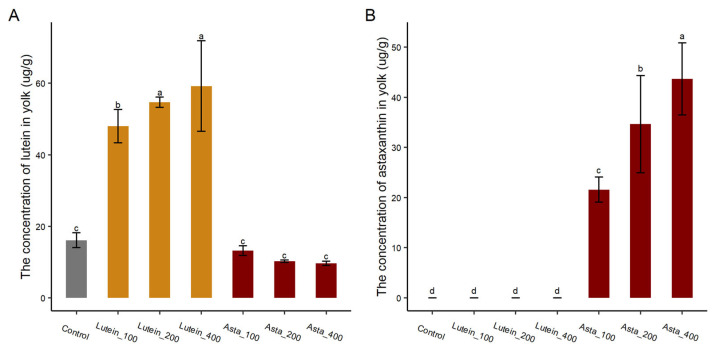
The (**A**) lutein and (**B**) astaxanthin content in egg yolk. ^a–d^ Bars with different letters mean significantly different, *p* < 0.05. Control: basal diet; Lutein_100, Lutein_200, and Lutein_400: basal diet supplemented with 100, 200, and 400 mg/kg lutein, respectively. Asta_100, Asta_200, and Asta_400: basal diet supplemented with 100, 200, and 400 mg/kg astaxanthin, respectively.

The content of the lutein and astaxanthin in egg yolk was analyzed. As shown in Figure 3, the lutein contents in egg yolk from the Lutein_100, Lutein_200, and Lutein_400 groups were 48.02, 54.71, and 59.20 μg/g, respectively, which were significantly higher than that in the control group (16.10 μg/g, *p* < 0.05). However, the lutein content in astaxanthin groups was lower than that in the control group, although the difference did not reach the statistical level (*p* > 0.05). Specifically, as astaxanthin supplementation levels increased from 100 to 400 mg/kg, the lutein content gradually decreased from 13.19 to 9.66 µg/g. This suggests that dietary astaxanthin may negatively influence lutein deposition. For astaxanthin, the contents were 21.57, 34.67, and 43.67 µg/g in Asta_100, Asta_200, and Asta_400 experimental groups, respectively. As expected, no astaxanthin was detected in the control group and lutein supplementation groups.

### 3.3. Lutein and Astaxanthin Supplement Influencing Carotenoid Deposition in Egg Yolk

To further assess the effects of lutein and astaxanthin supplementation on the deposition of other carotenoids in egg yolk, a quantitative profile of carotenoids was conducted via LC-MS. A total of 68 carotenoids were investigated, and only 26 constituents were discovered (Table S2). Among these, 14 constituents with a concentration greater than 0.1 μg/g were used for subsequent analysis (Table 3). In the control and lutein-supplemented groups, 5,6 epoxy-lutein-caprate-palmitate, lutein, and zeaxanthin were the three predominant constituents, accounting for 95% of the total carotenoid content. For the astaxanthin supplementation groups, astaxanthin, 5,6 epoxy-lutein-caprate-palmitate, lutein, and zeaxanthin were the four dominant carotenoids in egg yolk, accounting for over 82% of the total carotenoid content.

Dietary lutein and astaxanthin supplementation indeed influenced the deposition of various carotenoids in egg yolk. In the lutein-supplemented groups, in addition to lutein, there was a significant increase in the concentration of lutein oleate, lutein palmitate, and lutein stearate compared to the control group (*p* < 0.05). Additionally, a slight increase was also observed in the content of 5,6 epoxy-lutein-caprate-palmitate, capsorubin, and canthaxanthin, though they did not achieve significant levels (*p* > 0.05). On the contrary, the zeaxanthin concentrations in the three lutein groups were significantly lower than that of the control (*p* < 0.05). Moreover, as the dietary lutein levels increased, the zeaxanthin concentration decreased. In the Lutein_400 group, the zeaxanthin concentration was only 53% of that in the control group.

The astaxanthin content in yolk increased significantly with the supplementation of astaxanthin (*p* < 0.05), while the astaxanthin concentrations were almost the same in the Asta_200 and Asta_400 groups. In the astaxanthin-supplemented groups, a declining trend was observed in the concentration of most of the detected carotenoids. The concentrations of zeaxanthin, β-cryptoxanthin, and α-cryptoxanthin decreased significantly with the increase in dietary astaxanthin supplementation (*p* < 0.05). Meanwhile, the concentrations of lutein, 5,6 epoxy-lutein-caprate-palmitate, and lutein dipalmitate also presented a slight declining trend, though they did not reach a significant level (*p* > 0.05). In contrast, only the concentration of canthaxanthin increased significantly in the astaxanthin addition groups (*p* < 0.05). Compared to the control group, the total carotenoid concentrations significantly increased in astaxanthin supplementation groups (*p* < 0.05). However, when comparing with the lutein groups, the total carotenoid contents were still lower.

## 4. Discussion

Chicken eggs are considered one of the most indispensable and affordable sources of nutrients for humans. In addition to their high protein content, eggs are also rich in lipids (cholesterol, phospholipids, and triglycerides), which have an important impact on human health and the flavor of food [24,25]. Moreover, lipids in egg yolk serve as an effective vehicle for the absorption of egg-derived carotenoids in humans [26]. Notably, carotenoid-enriched eggs exhibit higher bioavailability compared to other dietary sources, such as supplements and spinach [27]. Therefore, chicken egg is a valuable vehicle to produce high bioavailability carotenoids.

In consumer acceptability tests, yolk color greatly influences consumer preferences [28]. As is known, yolk color is influenced by the amounts of carotenoids hens consume. Our results showed that supplementation with a high dose of lutein and astaxanthin significantly improved the yellowness and redness of egg yolk, respectively. Meanwhile, high dose of lutein and astaxanthin supplementation had no negative effects on eggshell strength, eggshell thickness, eggshell index, egg weight, albumen height, and Haugh unit. These results are consistent with the previous studies that dietary lutein and astaxanthin supplementation has no significant effects on egg quality except yolk color [16,29,30]. Carotenoid research has primarily concentrated on yolk color, with little attention to eggshell color. The eggshell color was evaluated by L*a*b* values, which represent lightness, redness, and yellowness, respectively. Lutein supplementation has no significant effects on L*, a*, and b* of eggshell. For astaxanthin supplemented groups, the L* value increased with the additional levels, suggesting that high doses of astaxanthin may potentially improve eggshell lightness.

Egg storability is an important indicator of egg shelf-life. Obviously, the albumen height and Haugh unit decreased significantly compared with fresh egg after one month of storage, while no significant effects on the albumen height and Haugh unit were observed among the seven groups. Egg moisture loss can lead to changes in egg texture and egg quality [31]. The egg weight loss of the Lutein_200, Lutein_400, Asta_100, and Asta_200 groups was significantly lower than that of the control group. Laying performance directly affects the economic benefits of poultry production. High doses of lutein and astaxanthin supplementation had no significant effect on laying rate, which is consistent with other studies [15,18,32,33].

Lutein content in egg yolk increased significantly as the dietary lutein supplementation increased. The same trend was also observed in astaxanthin addition groups. Our previous study showed that high-dose carotenoid supplementation reduces the transfer efficiency of carotenoids in egg yolk (unpublished data). Thus, while dietary high-dose carotenoids can rapidly increase the carotenoid content in egg yolk, they also raise the production cost of carotenoid-fortified egg. More importantly, we found that the concentration of lutein in yolk decreased with the increase in astaxanthin supplementation in the diet, suggesting that dietary astaxanthin might inhibit lutein deposition in egg yolk. To confirm this finding and further investigate the effects of lutein and astaxanthin supplementation on the deposition of other carotenoids, we analyzed the carotenoids in egg yolk using LC-MS. A total of 26 carotenoids were detected in egg yolk, including 1 carotene and 25 xanthophylls. Of these, 14 carotenoids had concentrations exceeding 0.1 μg/g, with 5,6 epoxy-lutein-caprate-palmitate, lutein, and zeaxanthin being the top three abundant carotenoids, accounting for 95% of the total carotenoid content in eggs; 5,6 epoxy-lutein-caprate-palmitate is a lutein ester that plays a role in plant photoprotection, pigmentation, and human nutrition [34]. Lutein and zeaxanthin, two important macular pigments, are widely recommended as dietary supplements for preventing age-related macular degeneration [35,36]. Despite the vast number of carotenoids, only about 20 carotenoids have been reported to be clearly identified in human blood and tissues [37]. Therefore, egg is an excellent source for obtaining lutein and zeaxanthin.

The LC-MS results revealed that the lutein concentration in yolk decreased with increasing dietary astaxanthin supplementation, confirming that astaxanthin supplementation competitively inhibits lutein deposition in egg yolk. Additionally, the concentration of zeaxanthin in yolk reduced with the increase in both lutein and astaxanthin supplementation, suggesting that both lutein and astaxanthin inhibit the deposition of zeaxanthin in egg yolk. Moreover, in the astaxanthin-supplemented groups, the concentration of lutein, 5,6 epoxy-lutein-caprate-palmitate, α-cryptoxanthin, and β-cryptoxanthin in egg yolks decreased as the levels of astaxanthin supplementation increased. This suggests that dietary astaxanthin might also inhibit the deposition of these compounds in egg yolk. Zhao et al. (2023) reported that dietary astaxanthin supplementation (approximately 60 mg/kg) decreased lutein content in egg yolks without affecting zeaxanthin [5]. Similarly, Wang et al., (2010) found competitive inhibition among lutein, zeaxanthin, and β-carotene in plasma and non-retinal tissues when the chickens were fed lutein (27.2 mg/kg), zeaxanthin (15.3 mg/kg), or beta-carotene (27, 58, or 227 mg/kg) [21]. These findings suggest that competitive inhibition occurs among different carotenoids during their deposition in egg yolk. However, the mechanisms underlying their competitiveness remain unclear and require further investigation.

On the contrary, the content of lutein palmitate and lutein oleate in yolk significantly increased with the addition of dietary lutein. The lutein powder used in this study was derived from marigold, which is considered as a good source for producing lutein-fortified eggs [38]. In addition to lutein, marigold flower contains a complex mixture of compound such as lutein monoesters, diesters, β-carotene, zeaxanthin, and violaxanthin [39]. Therefore, dietary lutein supplementation would not only increase the lutein content in egg yolk, but also increase the α-cryptoxanthin, lutein palmitate, and lutein oleate. The content of canthaxanthin in egg yolks increased with the additional levels of astaxanthin. The astaxanthin powder used in this study was derived from *Haematococcus pluvialis* [40]. The production of astaxanthin in algae can be affected by stress conditions. For example, under high light intensity or nutrient conditions, the carotenoid biosynthesis pathway is activated, resulting in the accumulation of canthaxanthin and other carotenoids in algae [41,42]. Therefore, dietary astaxanthin might also increase other carotenoids in egg yolk.

This study reveals the effects of high-dose lutein and astaxanthin supplementation on egg quality, laying performance, and carotenoid interaction. By optimizing the ratio of different carotenoids in feed, the competitive inhibition of carotenoid deposition in eggs can be reduced, and their bioavailability can be enhanced. In addition, functional eggs can be customized to contain varying carotenoid combinations. This provides an innovative vehicle for precision nutritional interventions. For instance, eggs can be designed with a specific ratio of lutein to zeaxanthin to enhance retinal protection for people at high risk of age-related macular degeneration.

## 5. Conclusions

In summary, supplementing high doses of lutein and astaxanthin in layer hen diets has no negative effects on laying rate and egg qualities, except for significantly improving yolk color. Twenty-six carotenoid components were detected in chicken egg yolk. Of these, 5,6 epoxy-lutein-caprate-palmitate, lutein, and zeaxanthin were the three most abundant carotenoids. High-dose lutein inhibited the deposition of zeaxanthin in egg yolk. What’s more, astaxanthin not only inhibited the deposition of zeaxanthin but also lutein and 5,6 epoxy-lutein-caprate-palmitate in egg yolk. Our study demonstrates that lutein and astaxanthin supplementation might induce competitive inhibition of carotenoids deposition in egg yolk. These findings provide guidance for the production of high-dose carotenoid-enriched eggs in the poultry industry.

## Figures and Tables

**Table 1:** Effects of dietary supplementation with lutein and astaxanthin on egg quality.

Item	Control Group	Lutein Supplementation Group			Astaxanthin Supplementation Group		
		Lutein_100	Lutein_200	Lutein_400	Asta_100	Asta_200	Asta_400
YC ^1^	9.13 ± 0.60 ^e^	10.5 ± 1.02 ^d^	11.0 ± 0.87 ^c^	11.4 ± 0.81 ^c^	16.57 ± 0.74 ^b^	17.68 ± 0.91 ^a^	18.01 ± 0.69 ^a^
ESS (kg/cm^2^)	3.98 ± 0.53 ^ab^	3.75 ± 0.65 ^b^	3.96 ± 0.79 ^ab^	3.80 ± 0.62 ^b^	4.24 ± 0.56 ^a^	4.24 ± 0.69 ^a^	3.83 ± 0.71 ^b^
EST (cm)	0.31 ± 0.03	0.30 ± 0.03	0.31 ± 0.02	0.31 ± 0.03	0.32 ± 0.03	0.31 ± 0.02	0.30 ± 0.02
ESC-L*	76.10 ± 4.50 ^abc^	75.95 ± 4.06 ^abc^	74.30 ± 5.21 ^bc^	75.22 ± 3.77 ^abc^	73.50 ± 11.64 ^c^	77.16 ± 3.72 ^ab^	78.10 ± 4.34 ^a^
ESC-a*	9.00 ± 2.49 ^ab^	9.53 ± 2.70 ^ab^	10.31 ± 2.44 ^a^	9.96 ± 2.32 ^a^	9.56 ± 3.93 ^ab^	9.09 ± 2.41 ^ab^	8.28 ± 2.85 ^b^
ESC-b*	20.05 ± 3.43 ^ab^	21.31 ± 3.17 ^ab^	21.73 ± 3.24 ^a^	21.43 ± 3.10 ^ab^	20.12 ± 5.62 ^ab^	20.39 ± 2.71 ^ab^	19.25 ± 3.31 ^b^
ESI	1.31 ± 0.04	1.31 ± 0.05	1.30 ± 0.04	1.31 ± 0.05	1.30 ± 0.04	1.31 ± 0.04	1.30 ± 0.04
EW (g)	45.3 ± 3.89 ^ab^	44.8 ± 3.13 ^b^	46.4 ± 3.49 ^ab^	45.3 ± 2.62 ^ab^	45.9 ± 3.54 ^ab^	46.8 ± 2.95 ^a^	46.5 ± 3.30 ^ab^
AH (mm)	4.01 ± 0.71	3.74 ± 0.89	3.82 ± 0.64	3.96 ± 0.76	3.86 ± 0.62	3.96 ± 0.72	3.94 ± 0.53
HU	66.1 ± 6.82	63.2 ± 10.7	63.8 ± 6.06	65.6 ± 7.55	64.5 ± 6.28	64.7 ± 6.97	65.1 ± 4.72

YC, yolk color; ESS, eggshell strength; EST, eggshell thickness; ESC, eggshell color; ESI, egg shape index; EW, egg weight; AH, albumen height; HU, Haugh unit. ^1^ The yolk color ranges from 1 to 19, where 1 represents bright yellow and 19 represents dark orange. L*, a*, b* present the lightness, redness, and yellowness of the eggshell, respectively. ^a–e^ Data with different superscripts within a row are significantly different at *p* < 0.05.

**Table 2:** Effects of dietary supplementation with lutein and astaxanthin on egg storability.

Item	Control Group	Lutein Supplementation Group			Astaxanthin Supplementation Group		
		Lutein_100	Lutein_200	Lutein_400	Asta_100	Asta_200	Asta_400
AH (mm)	2.57 ± 1.61	2.54 ± 1.29	2.32 ± 1.07	2.72 ± 1.30	2.63 ± 1.19	2.97 ± 1.53	2.61 ± 1.39
HU	51.04 ± 17.81	51.53 ± 14.97	49.45 ± 12.36	53.77 ± 14.72	51.77 ± 13.6	54.87 ± 15.76	50.76 ± 17.02

AH, albumen height; HU, Haugh unit.

**Table 3:** The carotenoid composition and content in egg yolk among different experimental groups (μg/g).

Compounds	Control Group	Lutein Supplementation Group			Astaxanthin Supplementation Group		
		Lutein_100	Lutein_200	Lutein_400	Asta_100	Asta_200	Asta_400
Lutein	13.67 ± 1.13 ^c^	43.96 ± 0.51 ^b^	51.18 ± 13.38 ^a^	54.02 ± 4.86 ^a^	11.34 ± 1.45 ^c^	10.13 ± 1.63 ^c^	7.02 ± 0.17 ^c^
Astaxanthin	ND	ND	ND	ND	19.58 ± 2.16 ^b^	30.37 ± 4.02 ^a^	29.08 ± 8.44 ^a^
5,6 epoxy-lutein-caprate-palmitate	14.31 ± 5.26	14.81 ± 1.19	16.14 ± 1.02	15.69 ± 0.84	14.56 ± 1.28	13.32 ± 1.83	12.99 ± 2.24
Zeaxanthin	11.75 ± 1.80 ^a^	9.55 ± 0.69 ^b^	8.05 ± 1.41 ^bc^	6.26 ± 1.43 ^cd^	7.45 ± 0.63 ^c^	6.35 ± 0.73 ^cd^	4.44 ± 0.26 ^d^
Violaxanthin-myristate-caprate	0.47 ± 0.14 ^ab^	0.38 ± 0.04 ^b^	0.36 ± 0.04 ^b^	0.47 ± 0.11 ^ab^	0.46 ± 0.06 ^ab^	0.48 ± 0.03 ^ab^	0.54 ± 0.06 ^a^
Lutein oleate	0.37 ± 0.05 ^c^	0.81 ± 0.05 ^b^	1.22 ± 0.09 ^a^	1.49 ± 0.49 ^a^	0.33 ± 0.09 ^c^	0.28 ± 0.07 ^c^	0.34 ± 0.05 ^c^
Capsorubin	0.32 ± 0.09	0.35 ± 0.06	0.35 ± 0.01	0.39 ± 0.12	0.33 ± 0.07	0.30 ± 0.03	0.30 ± 0.14
Violaxanthin myristate	0.31 ± 0.07	0.35 ± 0.09	0.38 ± 0.10	0.33 ± 0.07	0.35 ± 0.08	0.37 ± 0.01	0.29 ± 0.04
Lutein palmitate	0.18 ± 0.02 ^c^	0.25 ± 0.03 ^bc^	0.33 ± 0.03 ^ab^	0.37 ± 0.14 ^a^	0.17 ± 0.04 ^c^	0.18 ± 0.02 ^c^	0.23 ± 0.01 ^c^
Lutein dipalmitate	0.14 ± 0.02	0.12 ± 0.03	0.15 ± 0.04	0.15 ± 0.06	0.15 ± 0.06	0.13 ± 0.03	0.11 ± 0.01
β-cryptoxanthin	0.12 ± 0.01 ^ab^	0.12 ± 0.02 ^ab^	0.12 ± 0.02 ^ab^	0.11 ± 0.02 ^bc^	0.14 ± 0.01 ^a^	0.13 ± 0.01 ^ab^	0.09 ± 0.01 ^c^
α-cryptoxanthin	0.11 ± 0.00 ^bc^	0.12 ± 0.01 ^ab^	0.14 ± 0.02 ^a^	0.12 ± 0.02 ^bc^	0.09 ± 0.01 ^cd^	0.08 ± 0.01 ^de^	0.06 ± 0 ^e^
Lutein stearate	0.08 ± 0.00 ^b^	0.10 ± 0.02 ^ab^	0.11 ± 0.03 ^a^	0.12 ± 0.01 ^a^	0.11 ± 0.01 ^ab^	0.07 ± 0.01 ^b^	0.10 ± 0.02 ^ab^
Canthaxanthin	0.01 ± 0.01 ^d^	0.02 ± 0.00 ^d^	0.03 ± 0.00 ^d^	0.03 ± 0.01 ^d^	0.16 ± 0.02 ^c^	0.27 ± 0.02 ^b^	0.35 ± 0.03 ^a^
Total	41.79 ^e^	70.94 ^b^	78.56 ^a^	79.55 ^a^	55.22 ^d^	62.46 ^c^	55.94 ^d^

The carotenoids were measured by LC-MS, and only the compounds exceeding 0.1 µg/g are presented in this table. ND: not detected. ^a–e^ Data with different superscripts within a row are significantly different at *p* < 0.05.

## Data Availability

Data will be made available upon request.

## Supplementary Materials

The following supporting information can be downloaded at: https://www.mdpi.com/article/10.3390/ani15131869/s1, Table S1 Composition and nutrient levels of basal diet for laying hens (air-dry basis). Table S2 The content of carotenoids in egg yolk.

## Abbreviations

The following abbreviations are used in this manuscript:

YC	Yolk Color
ESS	Eggshell Strength
EST	Eggshell Thickness
ESC	Eggshell Color
ESI	Egg Shape Index
EW	Egg Weight
AH	Albumen Height
HU	Haugh unit
